# The emerging role of miR-20b in human cancer and other disorders: Pathophysiology and therapeutic implications

**DOI:** 10.3389/fonc.2022.985457

**Published:** 2022-12-13

**Authors:** Sheyda Khalilian, Hamid Abedinlou, Bashdar Mahmud Hussen, Seyedeh Zahra Hosseini Imani, Soudeh Ghafouri-Fard

**Affiliations:** ^1^ Student Research Committee, School of Medicine, Shahid Beheshti University of Medical Sciences, Tehran, Iran; ^2^ Department of Medical Genetics, School of Medicine, Shahid Beheshti University of Medical Sciences, Tehran, Iran; ^3^ Department of Medical Biotechnology, Faculty of Medicine, Kermanshah University of Medical Sciences, Kermanshah, Iran; ^4^ Department of Biomedical Sciences, Cihan University, Erbil, Kurdistan Region, Iraq; ^5^ Department of Pharmacognosy, College of Pharmacy, Hawler Medical University, Kurdistan Region, Iraq; ^6^ Division of Genetics, Department of Cell and Molecular Biology and Microbiology, Faculty of Biological Sciences and Technologies, University of Isfahan, Isfahan, Iran

**Keywords:** miRNA, miR-20b-5p, cancer, expression, biomarker

## Abstract

miR-20b is a microRNA with diverse and somehow contradictory roles in the pathogenesis of human disorders, especially cancers. It has been known to be a tumor suppressor in colon cancer, renal cell carcinoma, prostate cancer, osteosarcoma and papillary thyroid cancer. In lung cancer and breast cancers, both tumor suppressor and oncogenic effects have been identified for this miRNA. Finally, in T cell leukemia, hepatocellular carcinoma, esophageal squamous cell carcinoma and cervical and gastric cancers, miR-20b is regarded as an oncogenic miRNA. In several types of cancer, dysregulation of miR-20b has been recognized as a predictive marker for patients’ survival. Dysregulation of miR-20b has also been recognized in Alzheimer’s disease, diabetic retinopathy, myocardial ischemia/infarction, chronic hepatitis B and multiple sclerosis. In the current review, we have summarized the miR-20b targets and related cellular processes. We have also provided a review of participation of this miRNA in different human disorders.

## Introduction

MicroRNAs (miRNAs) have been initially discovered in *Caenorhabditis elegans*, but further experiments have identified them in most eukaryotes (1–3). These small non-coding RNAs have an average size of 22 nucleotides. The majority of miRNAs are transcribed from DNA sequences into primary miRNA transcripts which are then processed into precursor and mature miRNAs, respectively. In general, 3’ UTR of target transcripts is the main part of interaction between miRNAs and target mRNAs. This type of interaction mainly leads to suppression of expression of target transcripts (4). Yet, miRNAs can also interact with 5′ UTR, coding sequences, and promoters of selected genes (5). In addition, activation of gene expression has been reported to be an uncommon consequence of miRNAs interaction with target mRNAs (6). miRNAs have crucial functions in the regulation of developmental processes as well as pathophysiology of human disorders. The latter is best documented in the carcinogenic processes (7–9). In the context of cancer, several miRNAs have been found to contribute to the regulation of cellular activities leading to carcinogenesis as well as drug resistance (10).

In the current review, we have summarized the impact of a certain miRNA, namely miR-20b-5p in human disorders, especially cancer. miR-20b is encoded by *MIR20B* gene on Xq26.2. The precursor miRNA has two arms: miR-20b-5p and miR-20b-3p. These miRNAs have been shown to participate in the pathogenesis of a variety of malignant and non-malignant conditions. In the current review, we summarize the role of miR-20b-5p and miR-20b-3p in both conditions and explain its possible therapeutic implications. The reason for selection of miR-20b was its widespread dysregulation in several types of cancers, its tissue-specific effects and its contribution to the etiology of a number of non-malignant conditions which show its important roles in the cellular homeostasis. Moreover, the bioinformatics step revealed association of miR-20b with several important cellular pathways indicating importance of conduction of further research for identification of its roles in different disorders.

## Bioinformatics step

**Figure 1:**
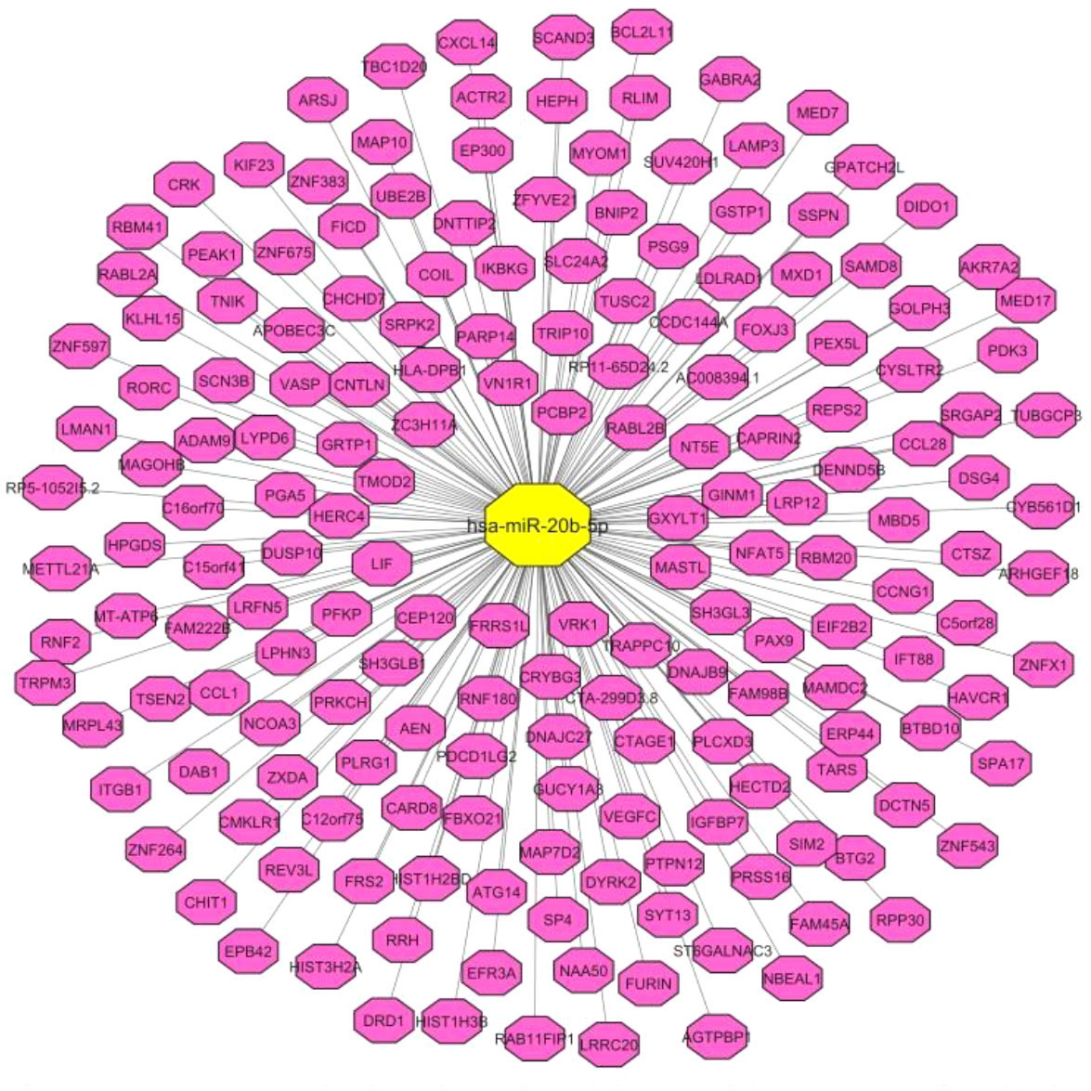
Correlation pairs of miR-20b-5p targets, predicted by TargetScan online tool. The interaction network was constructed by Cytoscape software.

First, through a bioinformatics step, we identified miR-20b-5p targets (**Figure 1**). Based on the results of TargetScan online tool (https://www.targetscan.org/vert_80/), miR-20b-5p is predicted to target a variety of mRNA targets being involved in a wide range of cellular functions.

**Figure 2:**
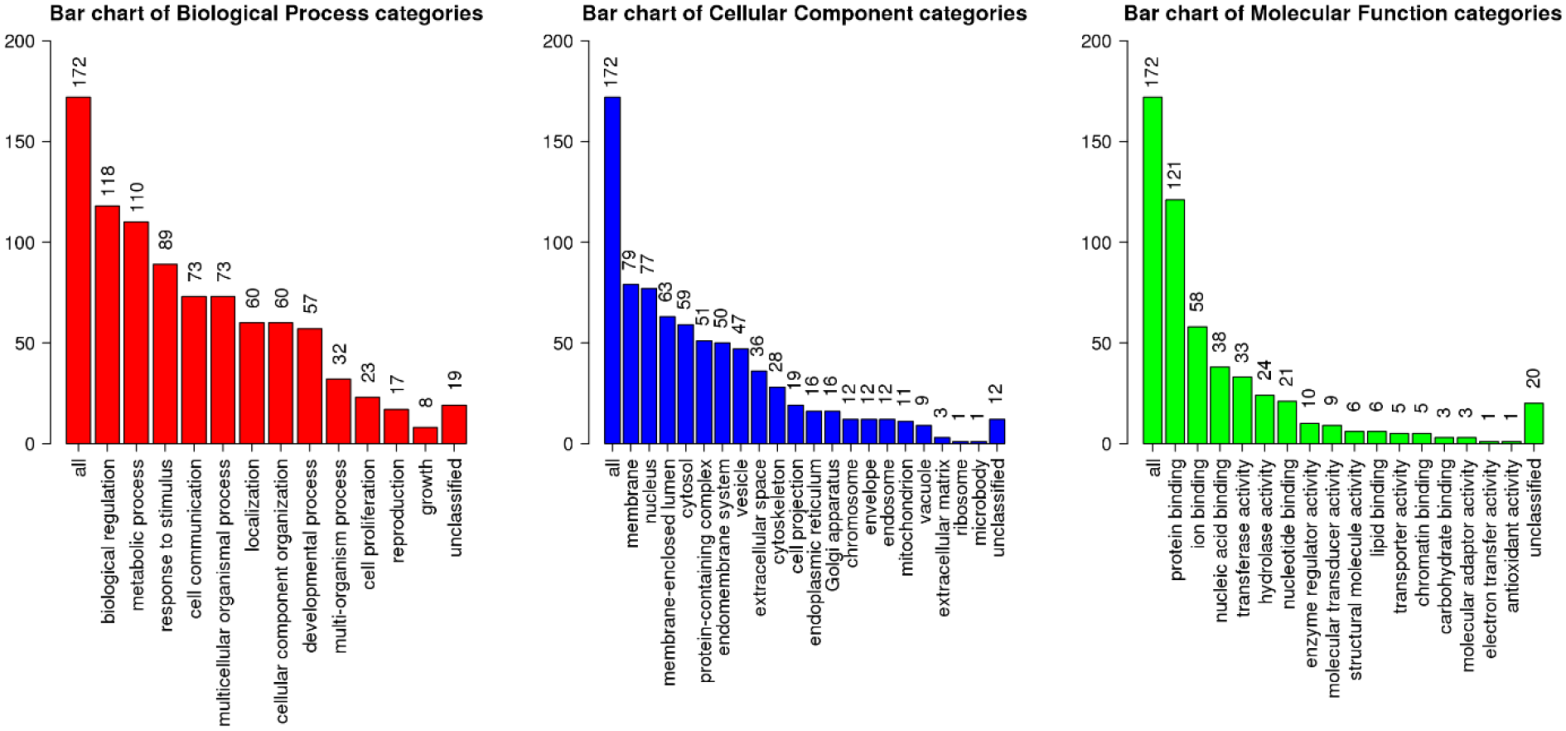
Functional enrichment analysis of top miR-20b-5p target genes, using WebGestalt database. The X-axis displays the name of different GO terms, and the Y-axis demonstrates the count of enriched genes. Besides, the length of the bar is showed according to the number of gene counts. GO, gene ontology.

Then, we identified the biological processes being influenced by this miRNA and its targets (**Figure 2**). Top target genes of miR-20b-5p are enriched in regulation of biological processes, metabolic processes, cell communication and developmental processes. Cell membrane and nucleus are top cellular components of miR-20b-5p targets. Finally, these targets are mostly implicated in protein binding, ion binding and nucleic acid binding.

## miR-20b role in cancers

Several studies have investigated the role of miR-20b-5p in the development or progression of colorectal cancer. Yang et al. have identified a tumor suppressor role for this miRNA in HCT-116 cell line. miR-20b-5p has been shown to inhibit cell cycle transition and reduce their migratory and invasive capacities without affecting cell apoptosis. Mechanistically, miR-20-5p tagets CyclinD1 (CCND1) transcripts in these cells. Up-regulation of miR-20b-5p has led to downregulation of CCND1 levels in HCT-116 cells. CCND1/CDK4/FOXM1 axis has been identified as the main route of participation of miR-20-5p in the pathoetiology of colorectal cancer. Besides, miR-20b-5p could inhibit tumorigenic effects of colorectal cancer cells in Balb/c nude mice (11). Another study in colorectal cancer cells has shown negative correlation between expression levels of miR-20b-5p and MALAT1. Both MALAT1 down-regulation and miR-20b-5p up-regulation could attenuate microsphere formation and self-renewal ability, decrease the proportion of cancer stem cells, and down-regulate levels of stemness-related proteins and those regulating cellular metabolism. The impact of si-MALAT1 and miR-20b-5p-mimic on suppression of tumorigenic abilities of HCT-116 cells has also been confirmed in xenograft model of cancer. Mechanistically, miR-20b-5p targets Oct4 in these cells (12). On the other hand, Ulivi et al. have reported up-regulation of miR-20b-5p circulatory level in metastatic colorectal cancer patients received bevacizumab in correlation with progression free and overall survival (13). Meanwhile, another study has revealed up-regulation of expression of the miR-20b-5p-songing lncRNA COL4A2-AS1 in colorectal cancer tissues and cell lines. In fact, COL4A2-AS1 could promote proliferation, and aerobic glycolysis of colorectal cancer cells *via* influencing the miR-20b-5p/HIF1A molecular route (14).

In lung cancer, miR-20b-5p has been identified as a tumor suppressor whose circulating serum exosomal levels could be applied as a diagnostic marker for early stage cancer (15). In this type of cancer, the tumor suppressor role of miR-20b-5p is mainly exerted through regulation of cell cycle transition and enhancement of cell apoptosis. Most notably, down-regulation of this miRNA has been associated with poor prognosis of adenocarcinomas and squamous cell carcinomas of lung (16). On the other hand, another study has reported up-regulation of miR−20b−5p in lung cancer cells. BTG3 has been identified as a target of miR−20b−5p. Up-regulation of miR−20b−5p enhances proliferation and migration of lung cancer cells (17).

**Figure 3:**
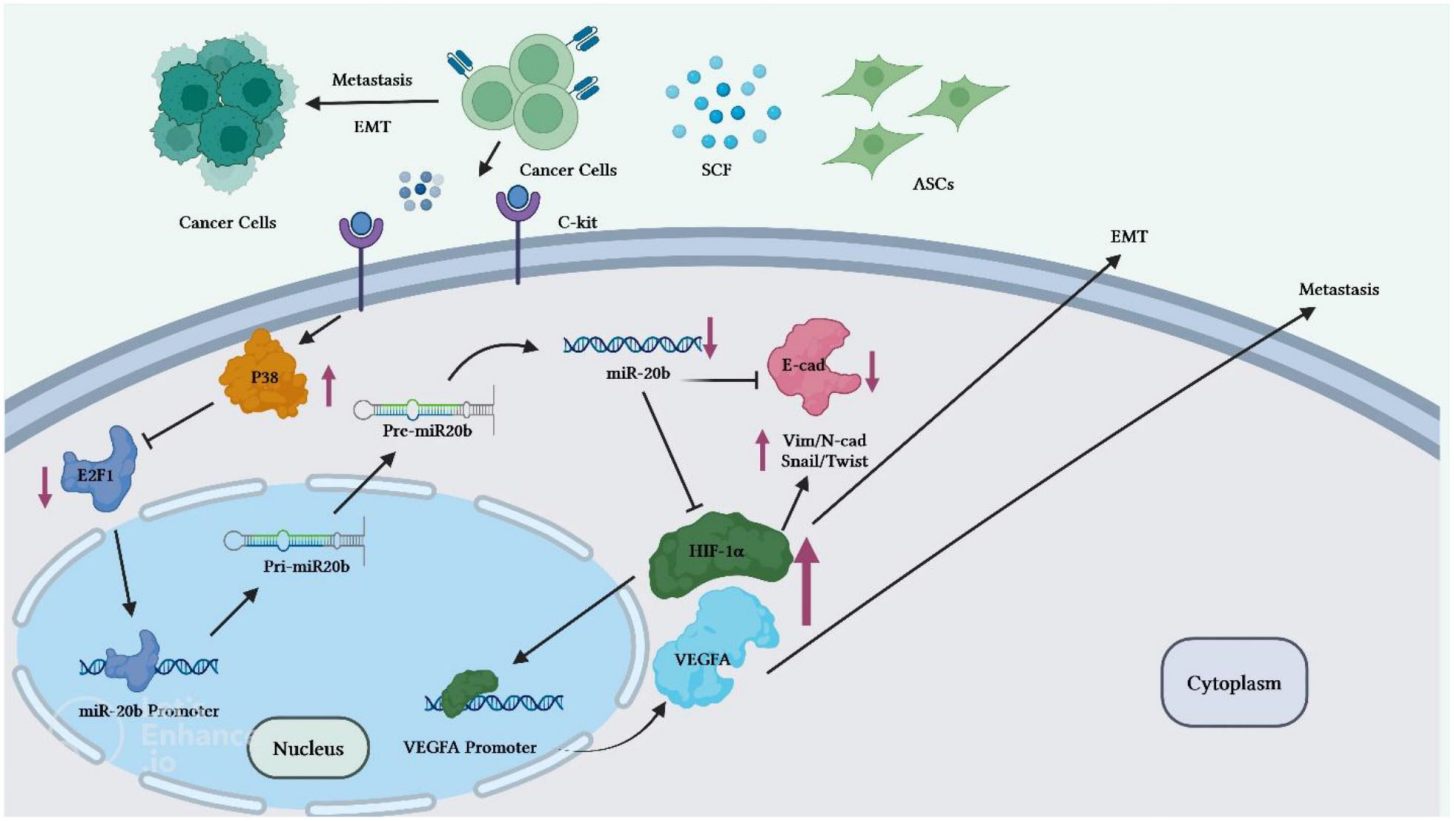
Overview of the potential breast cancer cells ASC-induced metastatic pathways. Through the regulation of miR-20b biogenesis, c-Kit-positive ASCs release high level of SCF to target BC cells. The MAPKp38/E2F1 pathway mediates the SCF-dependent decrease in miR-20b, which enhances activity of HIF-1/VEGFA and promotes EMT and metastasis of BC cells. Activation of the HIF-1α/VEGFA results in the upregulation of the N-cadherin, vimentin, and Twist expressions and decreased expression levels of E-cadherin. Therefore, through miR-20b suppression, ASCs induces metastasis of BC cells in animal models. ASC, Adipose tissue-derived mesenchymal stem cells, BC, Breast cancer cell.

Reduction of miR-20b level is also implicated in the progression of breast cancer. Adipose tissue-derived mesenchymal stem cells (ASCs) have been found to enhance breast cancer metastasis. Co-culture of breast cancer cells with these cells has led to enhancement of migration and invasive properties of breast cancer cells. Mechanistically, stem cell factor (SCF) produced by ASCs can reduce miR-20b biogenesis *via* regulation of c-Kit/MAPK-p38/E2F1 signaling. HIF-1α and VEGFA have been identified as targets of miR-20b. Down-regulation of miR-20b could activate HIF-1α-mediated VEGFA expression. On the other hand, over-expression of miR-20b has abolished epithelial-mesenchymal transition process and lung metastasis of breast cancer cells through suppression of N-cadherin, vimentin and Twist (18) (**Figure 3**).

**Figure 4:**
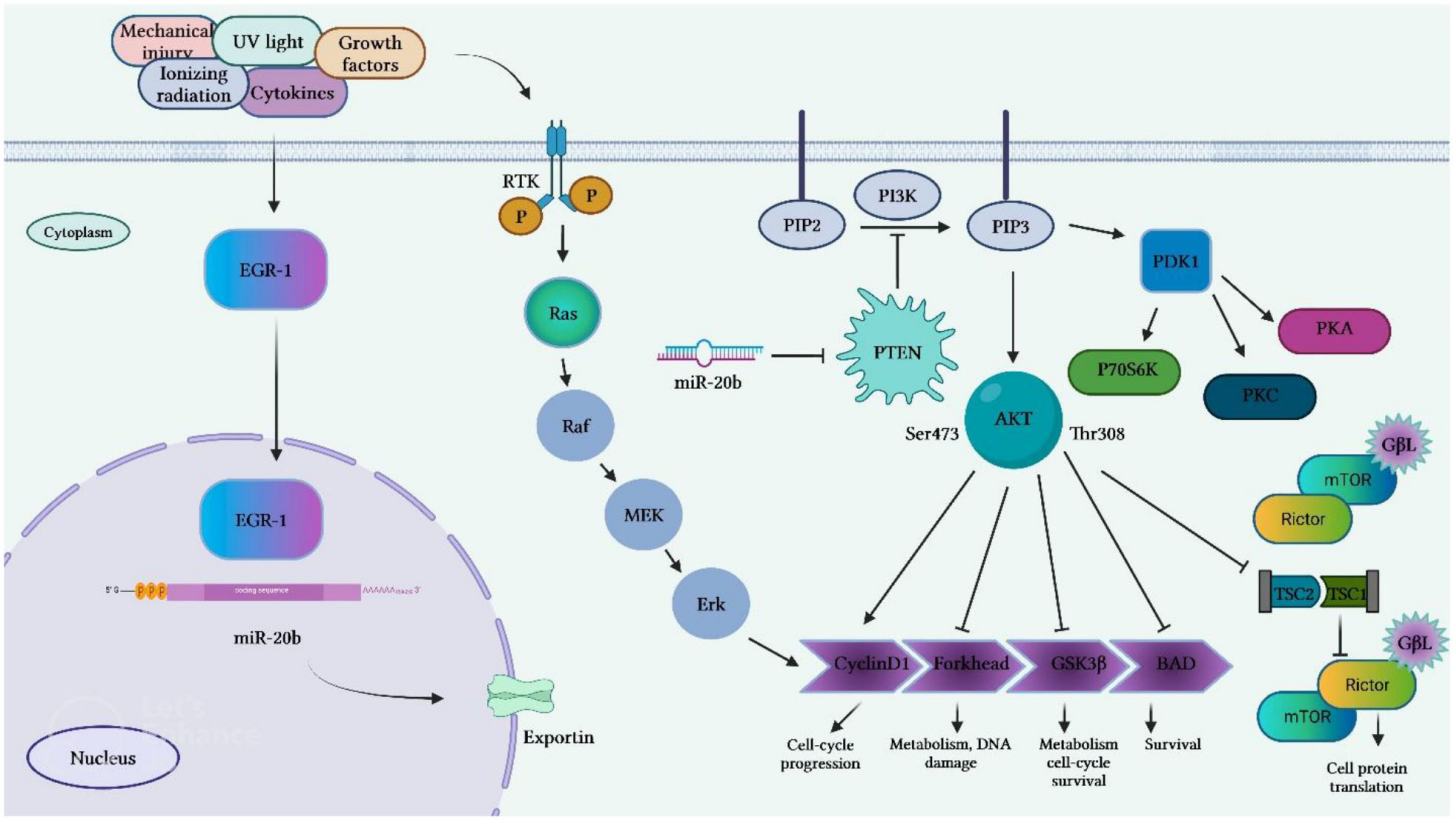
Transcriptional activation of miR-20b by EGR1 that leads to targeting PTEN. EGR1 is an important factor in transcription of miR-20b. EGR1 is triggered by numerous extracellular stimuli, including growth hormones, cytokines, ionizing radiation, ultraviolet light, and mechanical damage. After activation, EGR1 enters the nucleus and binds to the miR-20b promoter, resulting in the transcription of miR-20b. The mature miR-20b in collaboration with other constituents of RISC, which detects and binds to PTEN mRNAs induces suppression of PTEN expression, thus promoting cell proliferation and migration.

Li et al. have reported the presence of numerous GC-rich binding motifs for EGR1 in the promoter of miR-20b. Expression of this miRNA has been found to be increased by ionizing radiation and its over-expression has been correlated with expression levels of EGR1 in a certain breast cancer cell line. Expression of miR-20b has been decreased by siRNA-mediated silencing of EGR1. Inhibition of miR-20b expression has suppressed proliferation and migration of breast cancer cells and resulted in G0/G1 and S phase arrest. This miRNA has been found to target several tumor suppressor genes, including PTEN and BRCA1. Thus, inhibition of miR-20b has led to enhancement of PTEN and BRCA1 expression. Besides, miR-20b expression has been correlated with expression levels of EGR1 in breast cancer tissues (19). **Figure 4** shows transcriptional activation of miR-20b by EGR1.

**Figure 5:**
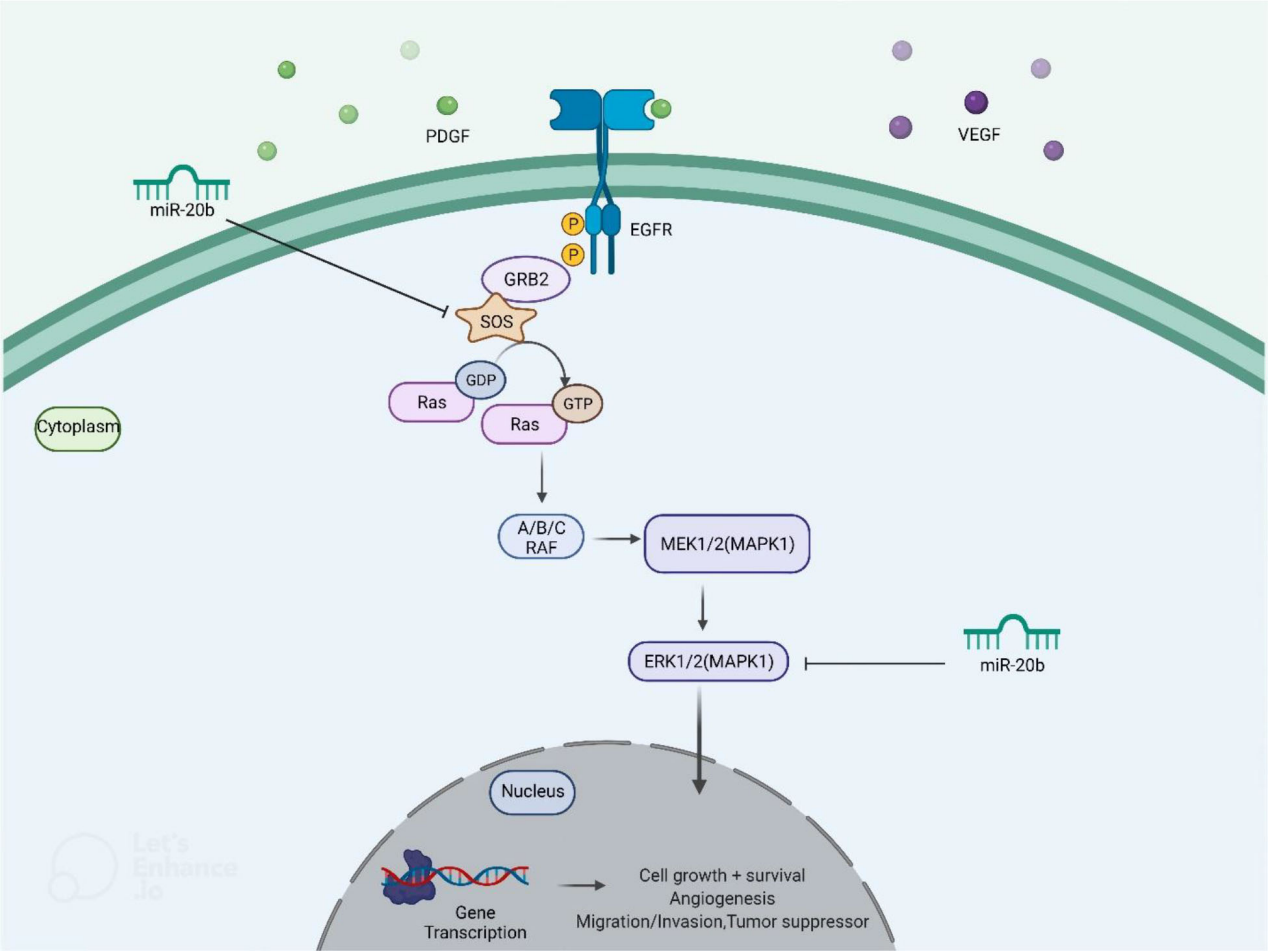
miR-20b- induced regulation of MAPK/ERK pathway. Through targeting SOS1 and ERK2, ectopic overexpression of miR-20b suppresses MAPK/ERK signaling cascade, leading to a reduction in cell initiation, progression, and metastasis.

miR-20b has been found to be down-regulated in papillary thyroid carcinoma tissues compared with paratumoral tissues. Notably, down-regulation of miR-20b has been associated with metastasis to cervical lymph nodes and TNM staging. Over-expression of miR-20b has suppressed viability, migration, and invasive properties of malignant cells and inhibited MAPK/ERK signaling *via* targeting SOS1 and ERK2. Besides, over-expression of miR-20b has suppressed growth of papillary thyroid carcinoma cells *in vivo* (20). **Figure 5** shows miR-20b- induced regulation of MAPK/ERK pathway in this type of cancer.

The impact of miR-20b in the pathogenesis of different types of cancers is shown in **Table 1**.

**Table 1:** Summary of the role of miR-20b in malignant conditions.

Type of cancer	ExpressionPattern	Samples	Cell line	Downstream targets	Pathway	Function	Kaplan-Meier	Ref
Colon Cancer	Down	-	HCT-116, SW480, and HT29, HIEC, 293 T cells, and 3T3 cells/female Balb/c nude mice	CCND1	MAPK, p53, VEGF	miR-20b-5p acted as a tumor-suppressor miRNA, and inhibited the cell cycle, migration, and invasion in HCT116 cells, through down-regulating CCND1	Over-expression of CCND1 has been associated with worse survival.	(11)
Colorectal Cancer (CRC)	Down	–	NCM460, COLO205, HCT‐116, LoVo, HT26, and SW480/nude Balb/c mice (n=24)	Oct4, GLUT1, LDHB, HK2, and PKM2	–	miR-20b-5p-attenuates self-renewal ability, reduced CSCs, and affected the stem cell-like properties of CRC cells.	–	(12)
Colorectal Cancer (CRC)	Up (upon bevacizumab-based treatment)	CRC patients peripheral blood samples (n=52)		RBL1		Circulating basal levels of hsa-miR-20b-5p predict clinical outcome of metastatic CRC treated with bevacizumab.	Longer progression-free and overall survival	(13)
Colorectal Cancer (CRC)	Down	55 pairs of CRC tumor tissues and adjacent normal tissues	T84, SW480, HT-29, LOVO, NCM460	HIF1A	–	miR-20b-5p is down-regulated by COL4A2-AS1 in CRC cells.	–	(14)
Colorectal Cancer (CRC)	Up (in SW480/5-FU cells compared with in SW480/WT cells)	BALB/c nude mice	SW480/5-FU	–	JNK/ERK	Interplay between miR-20b-5p and SDC2 induces proliferation of SW480/5-FU cells and their invasion.	–	(21)
Non-small Cell Lung Cancer (NSCLC)	Down	Peripheral blood of 276 patients and 282 controls	–	–	–	miR-20b-5p has a tumor suppressive role during the development of NSCLC and could be used as a biomarker for the diagnosis of early-stage NSCLC.	–	(15)
Non-small Cell Lung Cancer (NSCLC)	Down	GEO dataset	–	HMGA2, E2F7	–	miR-20b-5p has a tumor suppressive effect by regulating cell cycle and promoting apoptosis.	Patients with lower expression levels of miR-20b-5p tend to have worse OS in both lung adenocarcinomas and lung squamous cell carcinoma.	(16)
Non-small Cell Lung Cancer (NSCLC)	Up	113 pairs of tumor tissue and adjacent normal tissue samples	16HBE, A549, H1299	BTG3	–	miR-20b−5p promotes cell proliferation and migration *via* targeting BTG3.	Worse 5-years OS	(17)
Renal Cell Carcinoma (RCC)	Down	Serum samples of 110 RCC patients and 110 healthy controls, TCGA dataset (503 RCC patients)	–	ITGA4, NRP2	MAPK, Ras	miR-20b-5p inhibits cell proliferation, suppresses cell migration and promotes cell apoptosis.	–	(22)
Renal Cell Carcinoma (RCC)	Down	48 pairs of fresh RCC and adjacent normal tissue samples	786O, ACHN, and HEK293T	VEGFA, PAR-1, MAP3K8, CREB1	–	miR-20b-5p act as a tumor suppressor.	–	(23)
Prostate Cancer	Down	–	PC3, LNCaP, DU145, VCaP, and 22RV1/Male BALB/c nude mice, human(n=30)	TGFBR2	TGF-b1	miR-20b-5p suppressed migration and invasion of prostate cancer cells by increasing E-cadherin and decreasing vimentin.	–	(24)
Breast Cancer	Up	GEO dataset (GSE68271)	MCF-7, T47D/15 nude mice	CCND1, E2F1, MAPK, STAT3, R2b23b, RAB5BR, RABEP1, TAOK3, PPARDR and XIAP	–	miR-20b-5p acts as an oncogene.	–	(25)
Esophageal Squamous Cell Carcinoma (ESCC)	Up	92 pairs of frozen ESCC tissue and adjacent normal tissue samples/ serum of ESCC patients (n=102) and healthy controls (n=60)	TE1, EC109, KYSE30, KYSE150, KYSE180, KYSE450, KYSE510, and HEK293T	RB1, TP53INP1	AXL/HIF-1a	miR-20b-5p is an oncogene in ESCC.	Poor OS and prognosis	(26)
Chronic Lymphocytic Leukemia (CLL)	Did not differ significantly (between previously treated and untreated patient)	88 CLL patients	U-937	HIF1A, STAT3	–	miR-20b-5p overexpression has a significant prognostic role.	Low miR-20b-5p expression predicts poor OS in CLL.	(27)
Laryngeal Squamous Cell Carcinomas (LSCC)	–	Cancerous laryngeal tissue specimens (n=105)	–	–	–	Down-regulation of miR-20b-5p predicts risk of disease recurrence.	Low intratumoral miR-20b-5p expression has been associated with longer OS.	(28)
T-cell Acute Lymphoblastic Leukemia (T-ALL)	Up	Pediatric T-ALL cases (n=66), healthy unrelated bone marrow donors (n=5)	HEK293T DND-41, CCRF-CEM, Jurkat, BE-13, P12-Ichikawa and MOLT-4	PTEN, BIM	JAK-STAT, RAS	miR-20b-5p acts as an oncomiR in T-ALL.	–	(29)
Hepatocellular Carcinoma (HCC)	Up	40 pairs of HCC tissues and adjacent non-tumor samples	THLE-3 HepG2, LM3, Hep3B, Huh7, MHCC97H	–	TGF-β	Up-regulated WWOX-AS1has sponged miR-20b-5p, thus decreased cell proliferation, migration, and increased cell apoptosis.	–	(30)
Hepatocellular Carcinoma (HCC)	Up	TCGA-LIHC dataset (normal: n = 50, tumor: n = 375)	L-02, Huh-7, SK-Hep-1, HepG2, MHCC97H	CPEB3	–	miR-20b-5p enhances proliferation, migration and invasion.	–	(31)
Osteosarcoma	Down	46 pairs of OS tissue and matched normal tissue samples	HOS (TCHu167), MG-63 (TCHu124), hFOB1.19	BAMBI	–	miR-20b-5p upregulation inhibits cell proliferation.	–	(32)
Osteosarcoma	Down	35 pairs of cancer tissues and adjacent normal tissues	U2OS, MG-63, Saos-2, hFOB1.19	KIF23	–	miR-20b-5p inhibit the proliferation and migration *in vitro*, *via* targeting KIF23.	–	(33)
Papillary Thyroid Carcinoma (PTC)	Down	60 pairs of tumor tissues and the adjacent normal tissues	CGTH W-3, TPC1, Nthy-ori 3-1	DUXAP8, SOS1	MAPK/ERK	miR-20b-5p acted as a tumor suppressor in PTC and inhibits the progression of the disease.	–	(34)
Papillary Thyroid Carcinoma (PTC)	Down	47 pairs of PTC and normal tissue samples	K1, TPC1	SOS1, ERK2	MAPK/ERK	miR 20b displays tumor suppressor functions in PTC, by inhibiting the activity of MAPK/ERK.	–	(20)
Cervical cancer	Up	10 paired (normal/diseased) samples with CIN1, 2, 3 and *in situ* carcinoma (CIS), and 22 pairs of CIN2–3 and nearby normal tissues	–	–	–	miR-20b-5p could serve as a stratification biomarker to differentiate neoplastic and non-tumorous cases.	–	(35)
Gastric cancer (GC)	Up	–	AGS, MKN28	EREG, FAT4, IRF1, TXNIP, PTEN	–	miR-20b-5p is a promising biomarker.	–	(36)
Esophageal cancer (EC)	Up	–	KYSE-150R, KYSE-150 cells	PTEN	PTEN/PI3K/Akt	miR-20b-5p negatively regulates PTEN, and decreases apoptosis in radioresistant KYSE-150R cells.	–	(37)

Diagnostic role of miR-20b has been assesses in lung, esophageal and renal cancers (**Table 2**). Expression levels of this miRNA in peripheral blood or serum samples be used as diagnostic marker for these types of cancers with area under ROC curve values ranging from 0.78 (22) to 0.85 (26) in renal and esophageal cancers, respectively.

**Table 2:** Diagnostic value of miR-20b in cancers.

Type of disease	Number of samples	Distinguish between	Area undercurve	Sensitivity(%)	Specificity(%)	Ref
Non-Small cell Lung Cancer (NSCLC)	Peripheral blood specimens from 276 NSCLC patients and 282 healthy subjects	NSCLC patients vs. healthy controls	0.818	–	–	(15)
Renal Cell Carcinoma (RCC)	Serum from RCC patients (n=110) and healthy controls (n=110)	RCC patients vs. HC for testing set (n=140) RCC patients vs. HC for validation set (n=80)	0.807 0.780	–	–	(22)
Esophageal Squamous Cell Carcinoma (ESCC)	Serum from ESCC patients (n=102) and healthy controls (n=60)	ESCC patients vs. healthy controls	0.859	76.3%	87.1%	(26)

## miR-20b in non-malignant disorders

Expression of miR-20-5p has been shown to be speedily and stably decreased upon oxidative stress. H_2_O_2_ could hamper G1/S transition and suppress DNA synthesis. Up-regulation of miR-20b-5p could rescue cells from growth arrest. miR-20b-5p could regulate expressions of p21, CCND1 and E2F1. Oxidative stress could decrease expression of E2F1, in line with obstruction of G1/S transition and suppression of DNA synthesis and proliferation. The underlying mechanism includes autoregulatory feedback between miRNAs and E2F1 and E2F1 response to repair oxidative stress-associated DNA damage (38). Since oxidative stress is involved in the pathoetiology of several human disorders, particularly age-related pathologies, miR-20-5p represents an important biomarker and target for treatment of these conditions.

**Figure 6:**
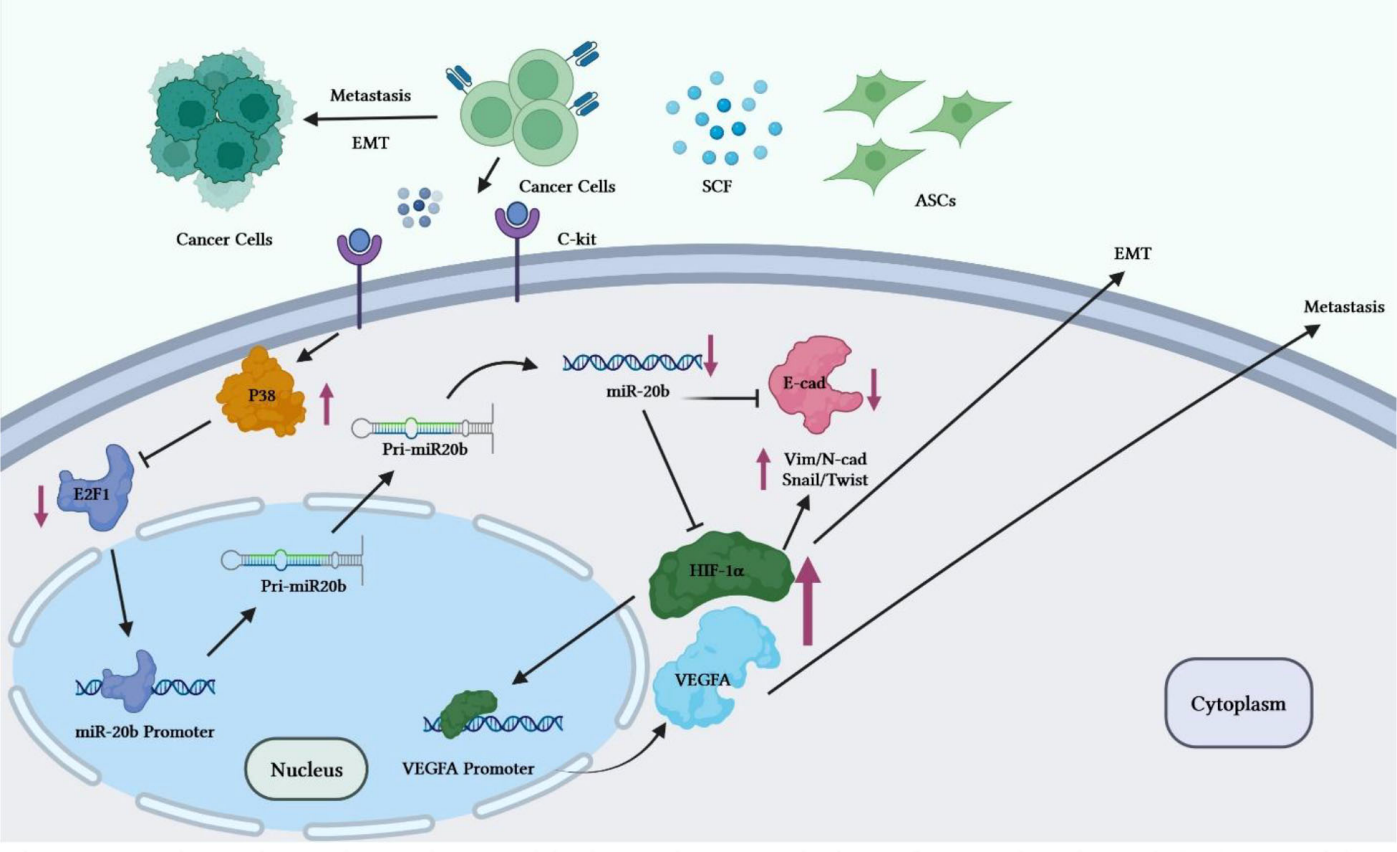
miR-20b-5p dependent and independent regulation of E2F1 levels and G1/S transition. The pathways I, II, and III as miRNA-dependent, and IV as miRNA-independent methods are proposed to modulate E2F1 levels under oxidative stress. In the pathways I and II, the lower expression of miRNAs leads to an increase in the p21 expression, and CCND1/2 and CDK4/6, respectively. In the pathway II, miRNA and E2F1 have an auto-regulatory feedback effect on each other. Finally, in the pathway IV, E2F1 is recruited to DNA break sites as a result of oxidative stress causing DNA damage responses. Overall, E2F1 is downregulated, the G1/S transition and DNA synthesis are inhibited, cell proliferation is repressed, leading to cell senescence. The proposed scheme is more fully described in the text.

**Figure 6** shows miR-20b dependent and independent regulation of E2F1 levels and G1/S transition.

Different study groups have assessed impact of miR-20b dysregulation in non-malignant conditions (**Table 3**). For instance, Wang et al. have reported participation of hsa-miR20b-5p in Alzheimer’s disease pathogenesis through regulation of Aβ. They have demonstrated alterations in miR-20b-5p level in association with progression of Alzheimer’s disease in three brain regions. miR-20b-5p could affect calcium homeostasis, neurite outgrowth, and branch points in human neurons *in vitro*. Notably, rs13897515 polymorphism of the *MIR20B* gene has been found to be associated with difference in Aβ1-42 levels in the CSF. Although miR-20b-5p has been shown to downregulate APP, it has been paradoxically associated with susceptibility to Alzheimer’s disease (39). Another study has shown that miR-20b-5p, *via* targeting RhoC gene, could disturb progression of Alzheimer’s’ disease by regulating the cell apoptosis, and cell viability (40).

**Table 3:** Summary of the role of miR-20b in non-malignant diseases.

Type of disease	ExpressionPattern	Samples	Cell line/Animal	Downstream targets	Pathway	Function	Ref
Alzheimer’s disease (AD)	Up	–	HeLa cells, U373MG/U373, HMC3, and SK-N-SH	APP	APP-mediated pathway	miR-20b suppresses APP by targeting the APP 3′-UTR. Increased miR-20b level results in loss of neuronal function.	(39)
	Up	–	PC12 cells/Animals (APPswe/PSΔE9) and control mice)	RhoC	–	miR-20b-5p, *via* targeting RhoC gene, could disrupt AD progression by regulating the cell apoptosis, and cell viability.	(40)
Diabetic Foot Ulcers	Up	10 control patients and 10 diabetes patients	HSF/C57BL/6 mice	VEGFA	WNT	miR-20b-5p suppresses wound repair by inhibiting the expression of VEGFA.	(41)
Diabetic retinopathy (DR)	Up	Retinal proliferative fibrovascular membranes from seven proliferative DR patients, Epiretinal membranes from six patients with idiopathic epiretinal membrane	Sprague −Dawley rats	BAMBI, TGF βR2, TCF7L2, PTEN, CDKN1A, BMPR2, ZBTB4	–	In diabetic conditions, upregulation of miR-20b-5p promoted HRMECs proliferation and migration, which result in formation of abnormal new vessels.	(42)
Type 2 diabetes (T2DM)	Up Up	10 T2DM patients, and 10 normal individuals 21 men with normal glucose tolerance, 16 men with impaired glucose tolerance, 21 men with type 2 diabetes	HUVECs/Male C57BL/6 mice HEK293 cells, HepG2 cells	Wnt9b STAT3, AKTIP, CYBRD1, TBC1D2, RNH1, GINM1, CFL2	wnt/β-catenin AKT	miR-20b-5p suppresses HUVEC angiogenesis *via* inhibition of the Wnt9b/β-catenin signaling pathway, therefore attenuates wound healing. Up-regulation of miR-20b-5p increased basal glycogen synthesis, and reduced AKTIP and insulin-stimulated glycogen accumulation.	(43) (44)
Myocardial Ischemia	Down	–	C57BL/6J mice	ATG7	–	miR-20b-5p suppresses autophagy and apoptosis of cardiomyocytes under hypoxia/reoxygenation conditions, therefore decrease the apoptotic death of cardiomyocytes.	(45)
Acute Myocardium Infarction (AMI)	Down	30 serum samples from acute myocardium infarction patients and 26 samples from normal persons	H9c2 cells	HIF-1α	NF-κB-p65	miR-20b-5p induces cardiomyocytes apoptosis under hypoxia conditions.	(46)
Chronic hepatitis B (CHB)	Up	liver puncture tissue in CHB patients complicated with Non-alcoholic fatty liver disease (NAFLD), and non-alcoholic steatohepatitis (NASH)	–	TXNIP	MALAT1/hsa-miR-20b-5p/TXNIP axis	miR-20b-5p/TXNIP axis may cause inflammatory damage in chronic HBV infection with NAFLD, and lead to the activation of NLRP3 inflammatory bodies and other inflammatory responses.	(47)
Ischemia-reperfusion (IR)	Up (in propofol- preconditioned HUVECS)	–	HUVECs	ULK1		Up-regulation of miR-20b-5p has a protective effect against hypoxia-reoxygenation (HR) injury, increasing cell viability and repressing autophagy and apoptosis.	(48)
Hepatic ischemia/reperfusion (I/R) injury	Down	–	Male C57BL/6 mice	ATG7	–	miR-20b-5p is sponged by HOTAIR, which attenuate its inhibitory effect on ATG7 expression, result in alleviating autophagy.	(49)
Tuberculosis (TB)	Down	72 TB patients and 60 normal volunteers	Forty adult male C57BL/6 mice	NLRP3	NLRP3/caspase-1/IL-1β pathway	miR-20b-5p alleviates the inflammatory responses *via* negatively regulation of NLRP3.	(50)
Multiple sclerosis (MS)	Up (upon natalizumab treatment)	17 patients with RR-MS	female SJL/J mice (n=5)	rorgt, stat3, and vegfa	NF-kB/MAPK	miR-20b has contributed in autoimmune demyelination *in vivo*.	(51)

miR-20b-5p is also involved in the pathogenesis of diabetic complications. Suppression of circulating exosomal miR-20b-5p has been found to accelerate diabetic wound repair (41). Another study has shown that down-regulation of the miR-20b-5p-sponging circular RNA circDMNT3B participates in the diabetic-associated dysfunction of retinal vessels (42). Moreover, inhibition of circulating exosomal miR‐20b‐5p could restore Wnt9b signals and reverse diabetes‐associated defects in wound healing (43). Therefore, this miRNA is a putative target for amelioration of diabetic complications.

## Discussion

miR-20b has been found to exert diverse and somehow contradictory roles in the pathogenesis of human disorders, especially cancers. It has been known to be a tumor suppressor in colorectal cancer (11), renal cell carcinoma (22), prostate cancer (24), osteosarcoma (32) and papillary thyroid carcinoma (20). In lung cancer and breast cancers, both tumor suppressor and oncogenic effects have been identified for this miRNA. Finally, in T cell leukemia (29), hepatocellular carcinoma (30), esophageal squamous cell carcinoma (37) and cervical (35) and gastric (36) cancers, miR-20b is regarded as an oncogenic miRNA. Such different roles of miR-20b might be related to tissue-specific targets of this miRNA. Based on our *in silico* approach, we demonstrated enrichment of miR-20b-5p targets in regulation of biological processes, metabolic processes, cell communication and developmental processes. Notably, these targets are mostly implicated in protein binding, ion binding and nucleic acid binding. Different levels of targets that are involved in binding with biomolecules in each tissue might affect the specific functions of miR-20b in these tissues.

Moreover, the precursor miRNA has two arms, namely -5p and -3p. Depending on the tissue or cell types, both arms might exert functional roles. Different functions of these arms might explain the tissue-specific roles of this miRNA.

In several types of cancer, dysregulation of miR-20b has been recognized as a predictive marker for patients’ survival. Although application of miR-20b as a diagnostic marker has only been investigated in lung, esophageal and renal cancers, the obtained results have been promising since serum/blood levels of this miRNA could separate cancer patients from healthy controls with acceptable diagnostic power. This finding potentiates this miRNA as a tool in non-invasive diagnostic approaches. Since this miRNA is dysregulated in several cancers, identification of abnormal levels of this miRNA does not point to a certain diagnosis. Instead it can be used for follow-up of patients after conduction of anti-cancer therapies for early diagnosis of cancer recurrence or metastasis.

Dysregulation of miR-20b has also been detected in Alzheimer’s disease, diabetic retinopathy, myocardial ischemia/infarction, chronic hepatitis B and multiple sclerosis. Wnt/β-catenin, AKT, NF-κB-p65, NLRP3/caspase-1/IL-1β and NF-kB/MAPK have been identified as the most important pathways being influenced by miR-20b in these conditions. Therefore, miR-20b-modulating therapies might affect course of these disorders through modulation of activity of these signaling pathways.

miR-20b has also been found to be sponged by a number of lncRNAs such as MALAT1, WWOX-AS1, PART1, DUXAP8 and COL4A2-AS1 as well as the circular RNA CircHIPK3. Thus, dysregulation of these non-coding RNAs might be regarded as a possible mechansim for alterations in the expression levels of miR-20b. Other putative mechanisms such as alterations in the epigenetic marks in the promoter of *MIR20b* should be assessed in future studies.

Taken together, miR-20b is a promising diagnostic marker in cancer and a putative therapeutic target in diverse human disorders. Future studies are needed to evaluate the efficacy of miR-20b-targeting strategies in animal and cell models of different disorders. In addition, the importance of this miRNA in modulation of response of cancer patients to chemotherapy, radiotherapy and targeted therapies should be assessed in future studies.

## Author contributions

Conceptualization, study design, investigation, validation of the collected papers, designing the tables and figures, review and editing were performed by SK; Figure preparation and completing the tables were performed by HA; Data collection and completing the tables were performed by SI; Draft manuscript preparation, revision and supervision were performed by SGF. All authors read and approved the final manuscript.
